# Guild Notes

**Published:** 1913-07-05

**Authors:** 


### *Guild Notes.*

**AN INVITATION.**

The Brighton meeting of the B.M.A. (July 18 to 26) will, no doubt, attract a very large number of medical men. The Guild has therefore arranged to open a temporary office there, and this will be at 13 Old Steine (next to the Old Steine Hotel).

One of the officials of the Guild will be constantly in attendance, and all possible information as to scope and organisation of the Guild will be available for visitors.

The afternoon will be the best time to come, and tea and refreshments will be served every day of the meeting.

**HEARD v. PICKTHORNE.**

This was a very important case for the medical profession. Dr. E., a member of the Guild, was not on the panel, but gave certificates to his private insured patients. These certificates were refused, on the ground that Dr. E. was not on the panel. The effect was to deprive our member of a large part of his practice, which lay almost entirely amongst the industrial classes. After taking the case to the Court of Appeal, the Guild obtained a judgment to the effect that the action of the friendly society involved was *ultra vires* and illegal. This precedent is, of course, good against any society in any part of the kingdom.

**CINDERFORD.**

Cinderford is the chief town of the Forest of Dean. The latter is a mining district, and the miners refused the demands of the medical men for 12s. a year per family—whatever the size—and set up Medical Aid Associations. The latter imported doctors to undercut the local resident practitioners. Here the Guild came in. It was most important to discover the status of the imported men, and, if possible, to get rid of them. It must be allowed that the imports themselves assisted a good deal in the attainment of the latter object. Without mentioning names, it may be stated that the inquiries of the Guild enabled them to ask the General Medical Council to send warning notices to more than one, and that the gentlemen so warned have now left. New ones are now being introduced, but from its knowledge of the facts the Guild feels that even if they get there they will soon be compelled to vanish again.

**MEDICAL AID ASSOCIATIONS.**

Steps have also been taken to put before the people who may become members of Medical Aid Associations the disadvantage of so doing, and the Guild is bringing to the notice of the Insurance Commissioners various irregularities proposed to be committed by these associations.

**THE GUILD AS A TRADE UNION.**

To any other body but a Trade Union the necessary steps might involve conspiracy or libel charges or damages for inducement to break contracts. Fortunately the Guild is a Trade Union. It is thus apparent how necessary it is that the profession should have a Trade Union at this juncture.

